# Thermal Insulation and Mechanical Properties of Polylactic Acid (PLA) at Different Processing Conditions

**DOI:** 10.3390/polym12092091

**Published:** 2020-09-14

**Authors:** Mohamed Saeed Barkhad, Basim Abu-Jdayil, Abdel Hamid I. Mourad, Muhammad Z. Iqbal

**Affiliations:** 1 Chemical and Petroleum Engineering Department, United Arab Emirates University, Al Ain 15551, Abu Dhabi, UAE; 200935051@uaeu.ac.ae (M.S.B.); mziqbal@uaeu.ac.ae (M.Z.I.); 2 Mechanical Engineering Department, United Arab Emirates University, Al Ain 15551, Abu Dhabi, UAE; ahmourad@uaeu.ac.ae

**Keywords:** polylactic acid, biopolymer, green thermal insulator, thermal properties, water retention, mechanical properties

## Abstract

This work aims to provide an extensive evaluation on the use of polylactic acid (PLA) as a green, biodegradable thermal insulation material. The PLA was processed by melt extrusion followed by compression molding and then subjected to different annealing conditions. Afterwards, the thermal insulation properties and structural capacity of the PLA were characterized. Increasing the annealing time of PLA in the range of 0–24 h led to a considerable increase in the degree of crystallization, which had a direct impact on the thermal conductivity, density, and glass transition temperature. The thermal conductivity of PLA increased from 0.0643 W/(m·K) for quickly-cooled samples to 0.0904 W/(m·K) for the samples annealed for 24 h, while the glass transition temperature increased by approximately 11.33% to reach 59.0 °C. Moreover, the annealing process substantially improved the compressive strength and rigidity of the PLA and reduced its ductility. The results revealed that annealing PLA for 1–3 h at 90 °C produces an optimum thermal insulation material. The low thermal conductivity (0.0798–0.0865 W/(m·K)), low density (~1233 $\mathrm{kg}/m^{3}$), very low water retention (<0.19%) and high compressive strength (97.2–98.7 MPa) in this annealing time range are very promising to introduce PLA as a green insulation material.

## 1. Introduction

Biodegradable materials have attracted much attention from researchers due to the pressing need to reduce enduring waste worldwide. Among the biodegradable materials, biodegradable polymers have received the most attention [1]. They are classified into three groups based on their sources: natural, semi-synthetic, and synthetic. Most biodegradable polymers are obtained from renewable sources such as starch. Aliphatic and mixed aliphatic/aromatic polyesters or polysaccharides (cellulose and its derivatives) are the most widely studied biodegradable polymers [2]. With depleting fossil-based raw materials for conventional polymers, biodegradable polymers provide an alternative with improved functional properties [3]. In addition, the biodegradability feature offers a real solution of municipal waste management issue.

**Figure 1:**
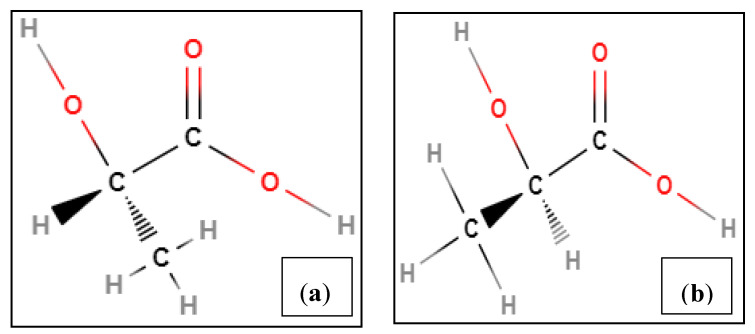
Chemical structure of (**a**) L-lactic acid and (**b**) D-lactic acid.

Polylactic acid (PLA) is a natural biodegradable polymer derived from renewable raw materials such as corn, starch, and sugarcane [4,5]. PLA is an enantiomeric polyester, which includes poly (L-lactic acid) and poly (D-lactic acid), as shown in Figure 1 [6]. Comparatively high D content (>20%) can yield an entirely amorphous polymer, whereas lower D content (<2%) leads to highly crystalline material [7].

Furthermore, PLA is a thermoplastic, high-modulus, high-strength polymer with large production rates and various applications. PLA is easily processable on standard plastic equipment into films, fibers or molded parts [5]. However, despite numerous positive features, PLA is brittle and rigid with low plastic deformation (~3%) [8]. The inherent brittleness shortens PLA use in implementations which require plastic deformation at higher levels of stress (e.g., fracture fixation plates and screws) [9].

PLA exhibits a slow degradation rate which is critical in biomedical applications [10] as well as in the disposal process of consumer commodities. The degradation of PLA is a function of its molecular weight and its distribution, crystallinity, morphology, stereoisomeric content and diffusion rate of water [11]. Nevertheless, PLA is relatively hydrophobic and lacks reactive side-chain groups on its surface [5,9].

The mechanical properties of a polymer are a complex function of a microstructure, such as chain relaxation, degree of crystallization and orientation [12]. Heat treatment (annealing) improves the mechanical characteristics of a polymer. Annealing improves heat and chemical resistance, glass transition and tensile strength in polymers [7,13]. Most likely, annealing improves a material’s performance by refining its crystallinity [7]. However, annealing may not alter melting and glass transition temperatures when the molecular weight is high enough [5,14].

The microstructural orientation and annealing are considered effective techniques for enhancing the mechanical performance of PLA. The orientation improves and increases toughness and strength whereas annealing improves the chain unwinding and crystallinity in PLA [12]. Temperature, shearing, stretching, and other processing conditions influence the resulting formation of the crystal structure [15]. PLA exhibits three crystalline forms: α, β, and γ, and a new crystal modification of α denoted as α′-form (disorder α) [15,16]. The α′-form appears by annealing PLA below 120 °C [16].

Takayama et al. [17] examined the mechanical properties of PLA/poly-epsilon-caprolactone (PLA/PCL) and PLA/poly-epsilon-caprolactone/Lysine triisocyanate (PLA/PCL/LTI) blends. These blends face annealing processes. The annealing impact on the PLA/PCL blend led to an increase in the flexural strength and modulus of it because of the crystallization of the PLA phase by annealing as well as the PLA/PCL/LTI blend which experienced polymerization besides annealing. In general, the annealing process leads to strengthening the microstructure, which consequently improves the mechanical properties.

Tabi et al. [18] reported effects of annealing PLA on crystallinity induced in neat, processed, and injection molded resins. They reported that annealing injection molding grade PLA in the temperature range of 100–140 °C for 10 min resulting in high crystallinity (up to 40%). Annealing PLA for 10 min in the 100–140 °C temperature range achieved very high crystallinity (40%) in an injection-molded grade PLA. Moreover, complete annealing disappeared the cold crystallization exotherm in PLA.

Weir et al. [19] investigated the effects of the annealing, molecular weight and morphology of poly-L-lactide (PLLA) on its degradability. The annealing increased crystallinity to 43% and 40% in compression-molded and extruded PLLA, respectively. Consequently, increased crystallinity decreased extension at the break and tensile strength whereas the Young’s modulus was improved.

Hassan et al. [20] added PLA to starch/cellulose composite foams to enhance their water barrier property and other properties. Different proportions of PLA were added to the mixture, and their thermo-mechanical and water absorption properties were analyzed. They found that PLA has a considerable impact in water barrier properties, flexural strength, and stiffness. Furthermore, the addition of PLA to the mixture increased the peak thermal degradation temperature resulting in the formation of a more stable structure.

PLA is the most extensively researched and utilized biodegradable aliphatic polyester in human history [5]. Due to its merits, PLA is a leading biomaterial for numerous applications in industry replacing conventional petrochemical-based polymers. Owing to its low thermal conductivity and hydrophobic nature, PLA can work as a potential thermal-insulating material to replace conventional fossil-based insulating materials. However, there is a scarcity of data on PLA as insulation material in the published literature to date. This work primarily focuses on the effects of processing conditions on the thermal insulation behavior of melt-processed PLA and its construction capacity. The impact of annealing conditions is reported on various thermal and mechanical properties as well as the morphology of PLA.

## 2. Materials and Methods

### 2.1. Materials

Poly(lactic acid) (PLA) (commercial grade 4032D; weight-average molar weight = $1\times 10^{5}$ g/mol; specific gravity = 1.24, and *T_m_* = 155–170 °C) was supplied by Zhejiang Zhongfu Industrial Ltd. and imported from Nature Works LLC (Hangzhou, China) in granule form with a ratio of L-lactide to D-lactide from 24:1 to 32:1 (manufacturer data). PLA was used in this study without any further modifications.

### 2.2. PLA Samples Preparation and Thermal Treatment

**Figure 2:**
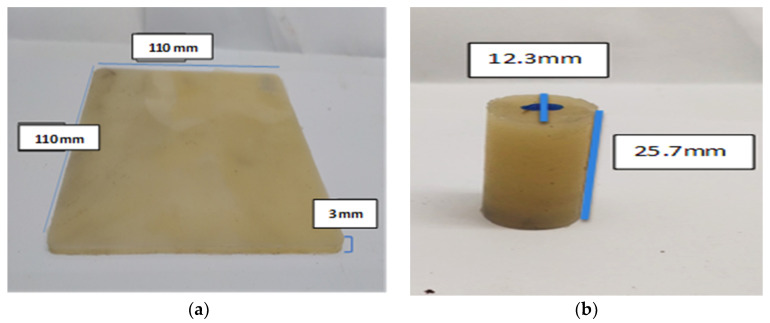
Dimensions of prepared specimens: (**a**) the thermal conductivity specimen; and (**b**) the water retention and compression strength specimen.

PLA was dried for two hours under vacuum at 90 °C followed by drying in a desiccator for one hour prior to processing due its hydrolytic susceptibility at higher temperatures. After that, an internal mixer mini-twin conical screw extruder (MiniLab Haake Rheomex CTW5, Dreieich, Germany) was used to melt the PLA pellets. PLA (5 g) was melt blended in a twin screw microcompounder (MiniLab Haake Rheomex CTW5, Dreieich, Germany) at 190 °C and 140 rpm for 3 min. The extruded PLA compression molded in hot press (Carver, AUTOFOUR/3015) in three heating-pressure cycles: (1) a force of 0.5 tons at 180 °C for 16 min, (2) a 0.52 ton force at 185 °C for 10 min, and (3) 3 tons of force at 100 °C for 3:30 min. The molded samples were thermally annealed in a convection oven (explained later). Figure 2 shows the molded sample for the thermal conductivity (a) and cylindrical sample (b) used for additional tests.

Thermal annealing was conducted in a convection oven at 95 °C. Since annealing between melting ($T_{m}$) and the glass transition temperature ($T_{g}$) is more effective [21,22], annealing was performed at 95 °C (PLA $T_{m}$ = 155–170 °C and $T_{g}$ ~ 60 °C). The annealing was conducted for a range of annealing times of 0.5, 1, 3, 10, 17, and 24 h. One sample was annealed “Fast” by cooling from hot press temperature to room temperature by flowing air over the samples in a fume-hood. Sample codes as a function of annealing time interval are provided in Table 1.

### 2.3. Scanning Electron Microscopy (SEM)

A JEOL-JCM 5000 NeoScope SEM (JEOL, Tokyo, Japan) was used to investigate the microstructure in processed PLA. The samples were sputter coated with gold for 3 min to remove the electrostatic charges in order to attain the maximum magnification of textural and morphological features. The SEM images are reported at various magnifications.

### 2.4. Thermal Conductivity

The thermal conductivity was measured using the Lasercomp FOX-200 thermal conductivity tester. Samples were shaped in specially designed molds with dimensions of 110 × 110 × 3 mm^3^ (Figure 2a) and thermal conductivity was measured using ASTM C1045-07 [23]. The results are reported as the average of three measurements.

### 2.5. Differential Scanning Calorimetry

**Figure 3:**
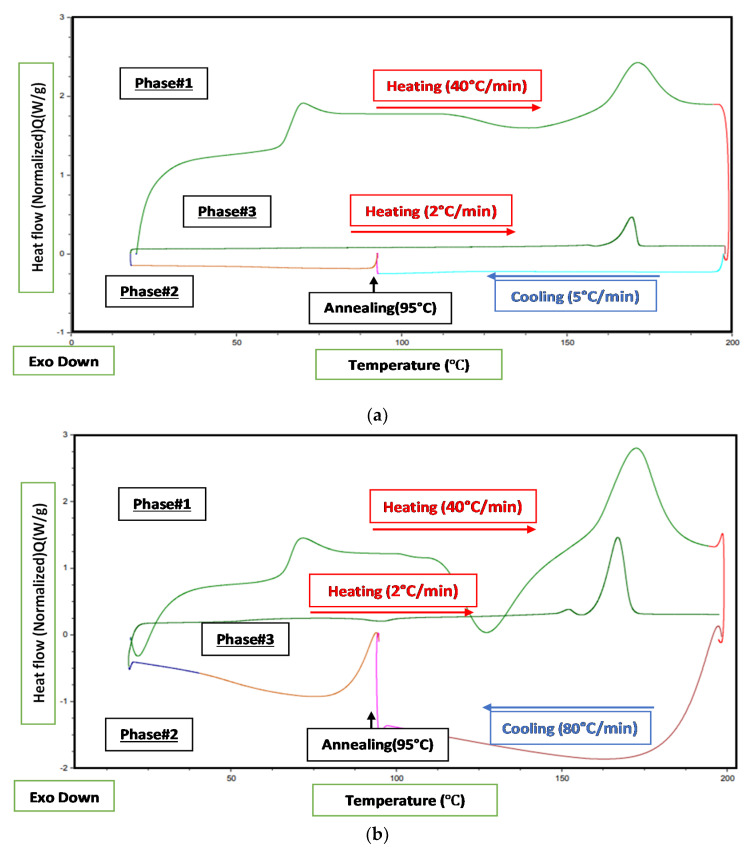
Differential scanning calorimeter (DSC) heating–cooling cycle for (**a**) Case A and (**b**) Case B.

Thermal properties of PLA were studied using a differential scanning calorimeter (DSC 25—TA Instruments, TA Instruments, New Castle, DE, USA). An approximately 5–7 mg sample was heated under a 50 mL/min nitrogen flow rate. The DSC results are reported as two cases: Case A simulates the oven-based annealing of PLA whereas in Case B, the effects of the fast cooling rate on PLA thermal properties are reported. The temperature protocols followed in these two cases are detailed below:

Case A: Samples were quickly heated to 200 °C at 40 °C/min and kept isothermally at 200 °C for 5 min to eliminate the thermal history. Subsequently, the molten samples were cooled to 95 °C at 5 °C/min and isothermally annealed for the following time intervals: 0 min (fast cooling/without stopping at 95 °C), 30 min, and 1, 3, 10, 17, and 24 h. This step is a simulation of the external annealing process. Following complete annealing, the samples were cooled to 20 °C at 5 °C/min and subsequently heated from 20 °C to 200 °C at 2 °C/min to evaluate the thermal characteristics (glass transition temperature and degree of crystallinity) as a function of annealing time interval (see Figure 3a).

Case B: Samples were quickly heated to 200 °C at 40 °C/min and kept isothermally at 200 °C for 5 min to eliminate thermal history (Figure 3b). The samples were rapidly cooled to 95 °C at 80 °C/min to avoid the development of premature crystallinity before reaching 95 °C. The samples were annealed at 95 °C for 0, 5, 10, 20, 30, 40, 50, or 60 min. Followed by annealing, the samples were cooled to 20 °C at 80 °C/min and subsequently heated from 20 °C to 200 °C at 10 °C/min to evaluate the thermal properties.

### 2.6. Compression Test

**Figure 4:**
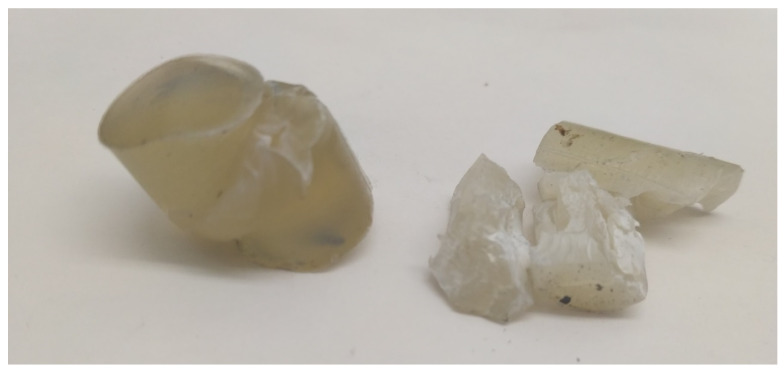
Samples after the compression test. On the left: buckled sample. On the right: fractured sample.

The compression test was conducted following ASTM D695-15 using a universal testing instrument (MTS model MH/20) with 100 kN load cell and with an overhead speed of 1.3 mm/min at room temperature. The compressive modulus, compressive strength, and elongation at break were reported. Cylindrical specimens of a length of 25.7 mm and diameter of 12.3 mm (Figure 2b) were compressed between the fixed (lower) and movable (upper) plates. The loading was continued until either the value of the load decreased by 10% of the maximum value or the specimen fracture occurred; otherwise, the experiment was interrupted manually when a specific contraction value was reached. Figure 4 shows the specimen after compression: the left image represents a buckled sample while the right one shows a fractured sample.

### 2.7. Water Retention

For the water retention test following ASTM D570-98 [24], two cylindrical specimens (L: 12.3 mm and D: 25.7 mm) (Figure 2b) were prepared for 0-PLA, 3-PLA, and 24-PLA. The samples were dried in an oven at 80 °C for four hours followed by drying in a desiccator until a constant weight was attained (called initial weight ($W_{initial}$)). The samples were immersed in distilled water at 25 °C. The specimens were removed within the first 24 h, pressed dry with a cloth and weighed up to seven times (Intervals: 3, 3, 3, 3, 4, 4, and 4 h). Within the second 24 h, each specimen was weighed up to five times (Intervals: 5, 5, 5, 5, and 4 h). Afterwards, the specimens were weighed once every three days. Total water absorbed was calculated using Equation (1):

(1) $$WR\left(\%\right)=\;\frac{W_{final}-W_{initial}}{W_{initial}}\;\times \;100$$

where *WR*(%) is the total water absorbed, and $W_{final}$ represents the weight after immersing in distilled water for a specific time. Three samples were tested for each type and averaged results were reported.

### 2.8. Density

The volume and the mass as a function of annealing intervals were measured up to four significant digits to calculate the sample density. The reported values are averages of three repetitions.

## 3. Results and Discussion

### 3.1. Thermal Properties of Processed PLA

#### 3.1.1. Case A

**Figure 5:**
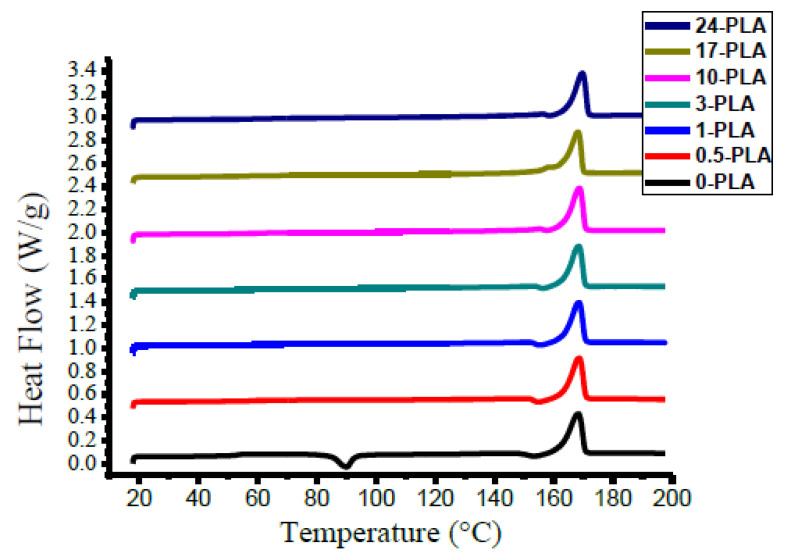
Effect of the annealing time on the DSC profile of processed polylactic acid (PLA).

The effect of the annealing time on the thermal behavior of processed PLA is shown in Figure 5. The DSC probes several properties of PLA including the glass transition temperature ($T_{g}$), melting temperature ($T_{m}$), crystallization temperature ($T_{c}$), enthalpy of crystallization ($\Delta H_{c}$), degree of crystallinity ($X_{c}$), and melting enthalpy ($\Delta H_{m}$). Table 2 lists the thermal properties extracted from the DSC profiles of the processed PLA.

Generally, increasing the annealing time of PLA increases $T_{g}$. The fast cooling sample exhibited $T_{g}$ = 53 °C whereas $T_{g}$ = 59 °C was observed in 24 PLA samples. The values of $T_{g}$ measured for neat PLA in this study were within the range reported in the literature [5,6]. In general, $T_{g}$ in PLA depends on D-lactate contents and the molecular weight. Accordingly, PLAs exhibit lower $T_{g}$ (up to 60 °C) compared to petroleum-based polyesters, with $T_{g}$ as high as 80 °C [14,25]. In addition, a cold crystallization exotherm was observed in the 0-PLA sample (Figure 5) at $T_{c}$ = 89.9 °C and $\Delta H_{c}$ = 19.2 J/g. The disappearance of the cold crystallization peak from annealed PLA samples (0.5-PLA–24-PLA) indicates that no more crystalline structures are generated during the heating cycle. There is a strong relationship between the annealing time and $\Delta H_{c}$, as when the annealing time increases, the $\Delta H_{c}$ decreases. In addition, almost there is no exothermic peak that could be identified at PLAs’ samples that have $X_{c}$ ~ 40% [18,26]. 

The annealing time showed no significant effect on the melting temperature as reported elsewhere [27]. The peak melting temperature slightly increased as a function of annealing time interval from 168.4 for 0-PLA to 169.7 °C for 24-PLA. However, increasing the annealing interval increased $\Delta H_{m}$, which was calculated as 50.6 J/g, 50.3 J/g, 54.5 J/g, and 60.3 J/g for 0-PLA, 3-PLA, 10-PLA, and 17-PLA, respectively. The increased $\Delta H_{m}$ further indicates a change in degree of crystallization with annealing. The degree of crystallization (%) was calculated by Equation (2):

(2) $$\%\mathrm{Crystallinity}\;=X_{c}=\;\frac{\Delta H_{m}-\Delta H_{c}}{\Delta H_{m}^{\infty }}$$

where $\Delta H_{m}$ is the experimental enthalpy of melting, $\Delta H_{c}$ is the enthalpy of cold crystallization if observed, and $\Delta H_{m}^{\infty }$ is the theoretical melting enthalpy of 100% crystalline PLA (93 J/g [28]). The degree of crystallization for all samples is reported in Table 2. The degree of crystallization improved substantially with the increasing annealing time. The calculated $X_{c}$ values were 33.8%, 51.3%, 54.1%, 58.6% and 64.8% for 0-PLA, 1-PLA, 3-PLA, 10-PLA, and 17-PLA, respectively. The 24-PLA exhibited a lower crystallization percentage indicating that PLA might have reached crystallization saturation between 17 and 24 h of annealing.

**Figure 6:**
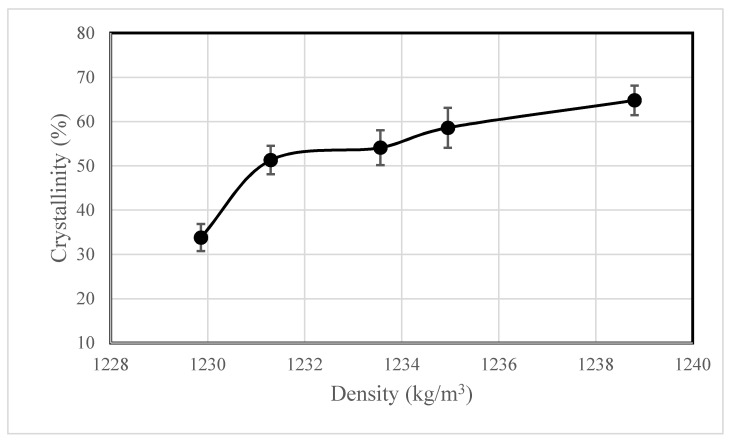
Degree of crystallinity versus samples density.

Figure 6 shows the degree of crystallization of the processed PLA and its measured density. With an increasing annealing time interval, the density increase was attributed to the structural compactness which consequently increased the degree of crystallization. It is noteworthy, however, that the density difference in 0-PLA and 17-PLA was not significant. The fast cooling in 0-PLA might not have resulted in a completely amorphous structure.

#### 3.1.2. Case B

**Figure 7:**
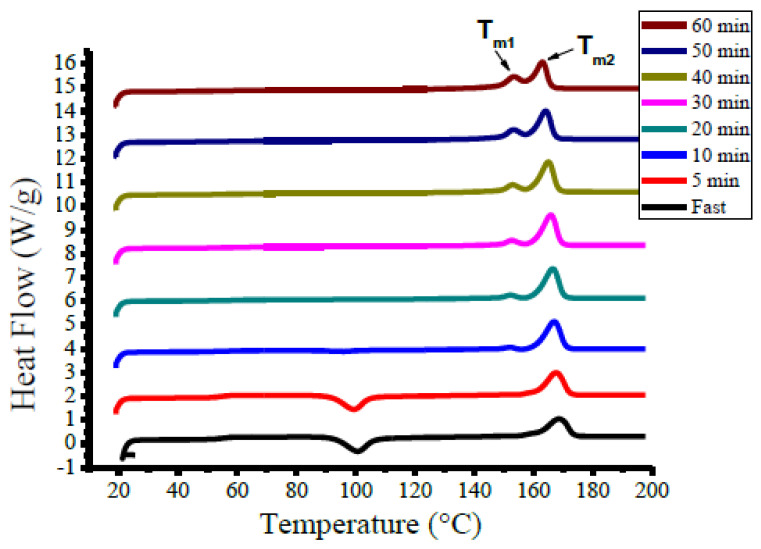
Effect of annealing time on the DSC profile of the processed PLA (Case B).

Case B involves the effects of fast cooling on the degree of crystallization ($X_{c}$) and the thermal properties of PLA before reaching the annealing temperature (95 °C). A cooling rate of 80 °C/min was applied to avoid any premature crystallinity before the annealing temperature (Figure 3b). The effect of annealing time (0, 5, 10, 20, 30, 40, 50 and 60 min) on the PLA’s thermal profile is shown in Figure 7 and the thermal parameters are enlisted in Table 3. The $T_{g}$ decreased slightly with the increasing annealing interval contradicting the behavior observed in Case A. In general, the observed $T_{g}$ was in the range of 49.1–55.1 °C [14,25]. Comparing $T_{g}$ with the annealing time, the fast cooling in Case B reduced $T_{g}$. Unlike in Case A, Figure 7 shows three peaks of crystallization at 101, 99, and 94.3 °C for a 0, 5, and 10 min annealing time, respectively. The appearance of these peaks indicated that these samples still have the capacity to produce crystalline structures during the heating cycle of the DSC.

Moreover, a shoulder to the melting point was observed for annealing over 5 min (Figure 7) attributed to the presence of two crystalline structures with annealing [15,16]. With increasing annealing time, two melting peaks ($T_{m1}$ and $T_{m2}$) appeared closer to each other (Table 3). The degree of crystallization increased with increasing annealing time following the Case A trend and similar to that previously reported by Angela et al. [29]. However, fast cooling in Case B produced a more crystalline PLA at the same annealing time.

### 3.2. Thermal Conductivity

**Figure 8:**
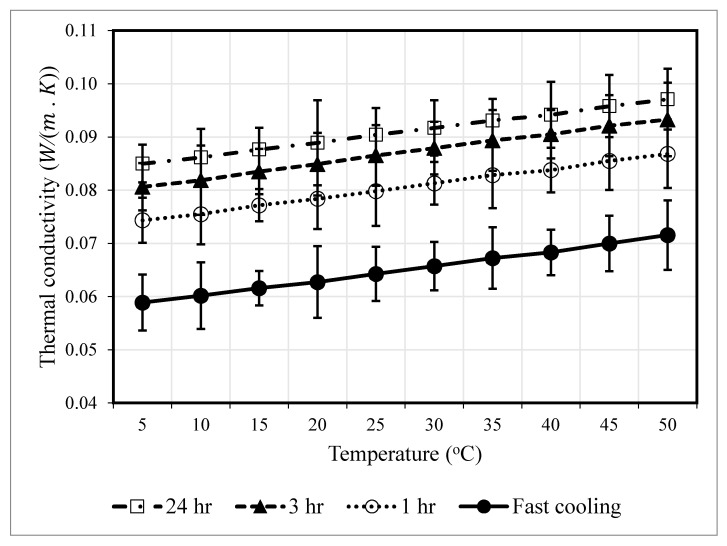
Thermal conductivity for PLA at different annealing times.

**Figure 9:**
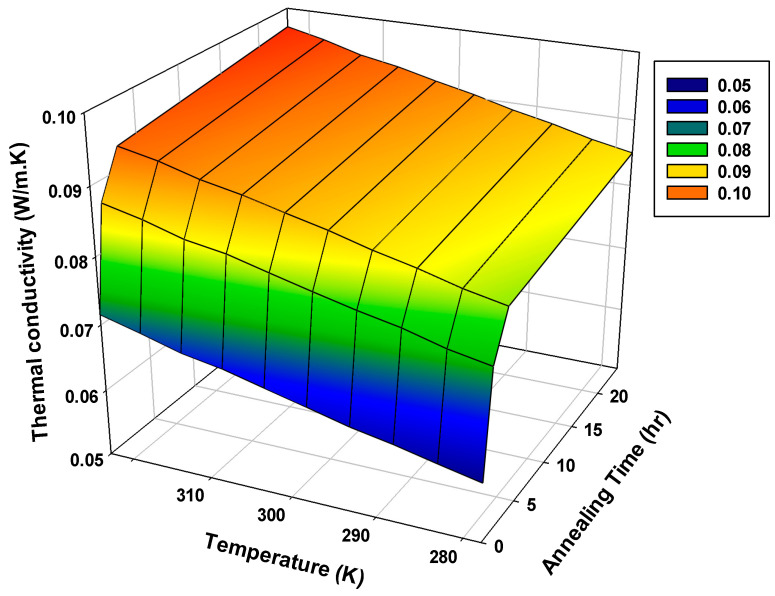
Dependence of the PLA thermal conductivity on the temperature and annealing time.

Figure 8 and Figure 9 show the effect of the annealing time on the thermal conductivity of processed PLA in the temperature range of 5–50 °C. A significant impact of the annealing process was observed on the thermal conductivity of PLA. For example, the thermal conductivity increased from 0.06426 W/(m·K) for the fast cooled PLA to 0.09044 W/(m·K) for the 24 h-annealed samples. Increasing annealing time interval increased the thermal conductivity. Increasing annealing time might have allowed spherulites to form comfortably and crystallites to grow in a more orderly fashion, which helped the fast heat transfer due to compact structure [22]. Additionally, the increasing degree of crystallization of PLA might have reduced gaps between the chains and air voids resulting in increased thermal conductivity.

The blue code in Figure 9 indicates the low thermal conductivity region where thermal conductivity is comparable to conventional insulators [30]. Since less thermal conductivity is desired, the samples annealed for one hour showed promising low thermal conductivities (0.074–0.086 W/(m·K)), indicating that they can act as thermal insulators.

### 3.3. Water Retention

**Figure 10:**
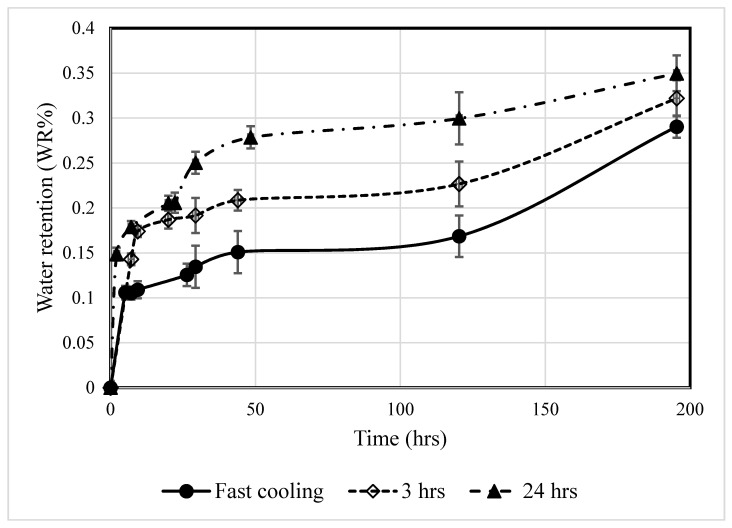
Water retention results for the processed PLA samples.

In the selection of thermal insulation materials, water retention is a pertinent physical property dictating the performance of thermal insulation materials. Figure 10 presents the effect of annealing time on the water retention (*WR*%) of processed PLA as a function of immersion time (hours). The WR% increased with increasing time without reaching an equilibrium value within 8 days of the immersion period. Furthermore, the WR(%) calculated from Equation (1) increased with the annealing time (24 h > 3 h > fast cooling).

It is difficult to compare the water retention results from this study with the published literature due to the scarcity of published data. The processed PLA exhibited a very low *WR*% (<0.35%), which is less than polyesters and polyester-based composites [31], and various types of polyurethane (1–6% after 24 h) [32] proposed for thermal insulation applications. The low *WR*% of PLA is attributed to its hydrophobic characteristics [33] which make it a potential candidate for thermal insulation.

### 3.4. Mechanical and Morphological Characteristics

**Figure 11:**
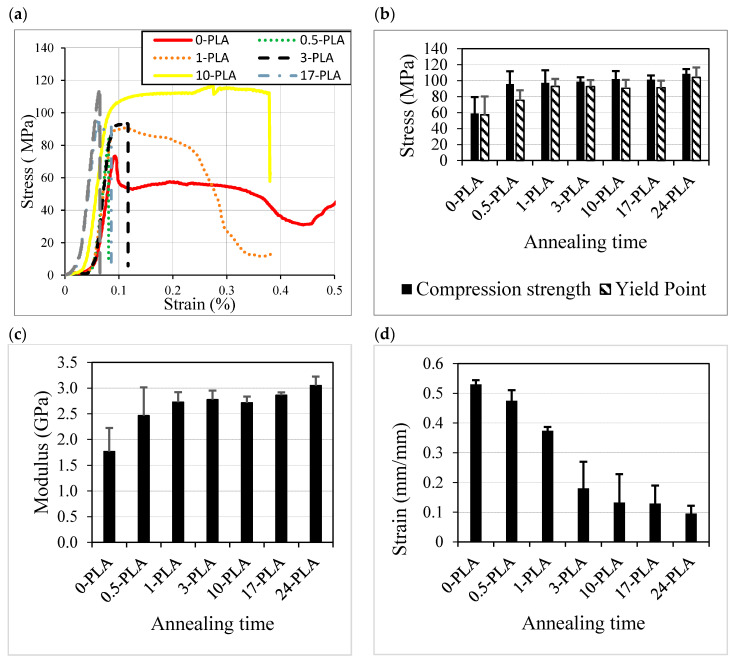
Effect of the PLA annealing time on (**a**) the stress–strain curve, (**b**) the compressive strength, and yield point, (**c**) the compressive modulus and (**d**) the compressive strain.

The mechanical performance of semi-crystalline polymers depends on multiple factors including the degree of crystallinity. The stress–strain behavior of processed PLA is shown in Figure 11a. The effect of the annealing process on the compression properties of PLA is presented in Figure 11a–d.

Increasing the annealing time improved the compressive strength, compressive yield point, and the compression modulus of the processed PLA. The compressive strength of the fast-cooled PLA was 58 MPa, which increased by 70% and 84% to reach 98.7 MPa and 108 MPa upon annealing for 3 and 24 h, respectively. This behavior can be attributed to the shaping of the spherulites and crystals during the annealing period. Therefore, increasing $X_{c}$ is likely to strengthen the structure of the polymer, resulting in the increase in the compressive strength and compressive modulus [17]. The compressive modulus increased with the annealing time interval, reaching a maximum for 24-PLA (Figure 11c). The 24 h-annealed PLA exhibited a 73% increment compared to the neat PLA, from 1700 MPa to ~3000 MPa.

Figure 11d shows that the maximum strain decreased with the annealing time interval. A maximum strain decrease was noticed for the 24-PLA where the maximum strain reduced from 0.58 for 0-PLA to ~0.095 for 24-PLA. The annealed PLA samples gradually lost their ability to absorb energy to fracture with the increasing annealing time interval. Moreover, the decreased fracture strain also indicates the formation of a more compact structure with annealing, in agreement with above results. The length of the annealing process plays a pivotal role in rearranging the PLA’s microstructure as reflected by the mechanical properties. The annealing process reduces the degree of molecular randomization and hence, improves the mechanical modulus and overall strength [22]. Overall, the PLA annealed from 1 to 3 h produces high compressive strength PLA (97.2–98.6 MPa) with a high degree of ductility (strain ~ 0.37–0.18%). The compressive strength and modulus were high compared to the polyester and its composites (strength: 19–103 MPa and modulus: 264–1370 MPa) [34] used for insulation applications.

**Figure 12:**
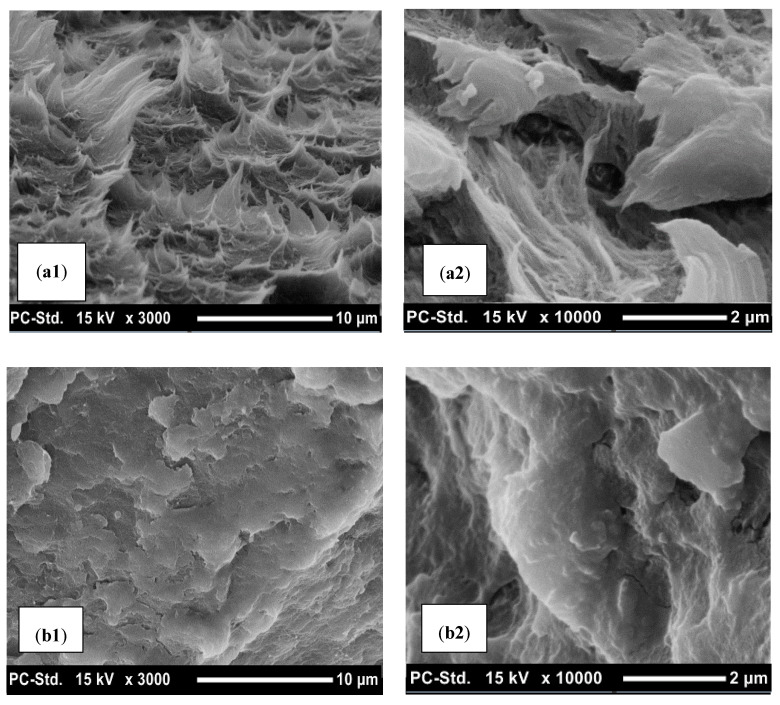
SEM micrograph for the PLA specimens at different resolutions: (**a1**) 0-PLA (low zoom), (**a2**) 0-PLA (high zoom), (**b1**) 24-PLA (low zoom), and (**b2**) 24-PLA (high zoom).

The SEM micrographs of the annealed PLA shown in Figure 12 support the observed mechanical and thermal behavior. Figure 12A1,A2 show the morphology of 0-PLA at 10 μm and 2 μm, respectively. However, it can be described as ductile, while Figure 12B1,B2 exhibit the morphology of 24-PLA at 10 μm and 2 μm, respectively, which can be recognized as brittle. The comparison between the 0-PLA and the 24-PLA samples revealed that the ductile deformation of the 0-PLA is suppressed by the annealing and the microstructure of 24-PLA is more rigid than that of 0-PLA. The voids and cavities between the polymer segments were reduced significantly upon annealing for 24 h.

## 4. Conclusions

**Figure 13:**
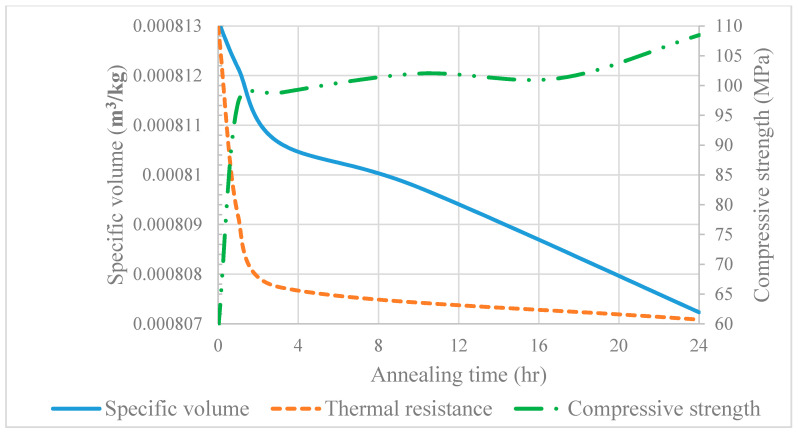
Thermal resistance, specific volume, and the compressive strength of PLA.

In this study, the effect of the annealing conditions on the thermal insulation and mechanical properties of the biopolymer PLA processed with a melt extruder and shaped in a compression molding system was reported. Increasing the annealing time in the range of 0–24 h led to a significant increase in the degree of crystallization, which had a direct effect on the thermal conductivity, density, and glass transition temperature. The thermal conductivity of PLA at 25 °C increased from 0.06426 W/(m·K)) for the fast-cooled samples (0-PLA) to 0.09044 W/(m·K)) for the samples annealed for 24 h (24-PLA). The glass transition temperature increased approximately by 11.33% between the 0-PLA and the 24-PLA samples to 59 °C. Moreover, the annealing process improved the compressive strength and rigidity of PLA substantially, by more than 80%, and reduced its ductility. From a thermal insulation perspective, the low thermal conductivity, low density, and high compressive strength are required. A brief comparison between the aforementioned properties is presented in Figure 13 and shows that the annealing process of PLA for 1–3 h produces an optimum thermal insulation material. The PLA thermal conductivity values (0.0798–0.0865 W/(m·K)), and density (~1233 $\mathrm{kg}/m^{3}$) in this annealing time range are very promising and comparable with traditional and commercial heat insulators, while the corresponding compressive strength (97.2–98.7 MPa) is much higher than that of traditional thermal insulators and comparable with construction materials.

## Figures and Tables

**Table 1:** Samples abbreviation.

	Annealing Processing Interval
0-PLA	Fast
0.5-PLA	0.5 h
1-PLA	1 h
3-PLA	3 h
10-PLA	10 h
17-PLA	17 h
24-PLA	24 h

**Table 2:** Effect of annealing time on the DSC profile of the processed PLA.

Interval (h)	$T_{g}$ **(°C)**	$T_{c}$ **(°C)**	$T_{m}$ **(°C)**	$\Delta H_{c}$ **(J/g)**	$\Delta H_{m}$ **(J/g)**	$X_{c}$ **(%)**
Fast (0)	53	89.9	168.4	19.2	50.6	33.8
0.5	57.6		168.7		48.8	52.5
1	56		168.6		47.7	51.3
3	56.4		168.5		50.3	54.1
10	57.6		168.8		54.5	58.6
17	56.6		168.3		60.3	64.8
24	59		169.7		54.3	58.4

**Table 3:** DSC results for the PLA in one hour of the annealing process at 95 °C (Case B).

Interval (min)	$T_{g}$ **(°C)**	$T_{c}$ **(°C)**	$T_{m1}$ **(°C)**	$T_{m2}$ **(°C)**	$\Delta H_{c}$ **(J/g)**	$\Delta H_{m1}$ **(J/g)**	$\Delta H_{m2}$ **(J/g)**	$X_{c}$ **(%)**
Fast (0)	55.1	101	168.6		32.5	36		38.7
5	54.3	99	167.6		30.4	41.4		44.5
10	54	94.3	152	167	3.5	2.5	43.6	49.6
20	55		152.5	165.8		3.8	40.9	48.1
30	54.1		152.8	165		5.3	36.3	44.7
40	52.1		153.1	164.2		54.7		58.8
50	50.8		153.2	163.1		55.4		59.6
60	49.1		153.3	162.1		55		59.1
